# When the Choice Is Ours: Context and Agency Modulate the Neural Bases of Decision-Making

**DOI:** 10.1371/journal.pone.0001899

**Published:** 2008-04-02

**Authors:** Birte U. Forstmann, Uta Wolfensteller, Jan Derrfuss, Jane Neumann, Marcel Brass, K. Richard Ridderinkhof, D. Yves von Cramon

**Affiliations:** 1 Amsterdam Center for the Study of Adaptive Control in Brain and Behaviour, Universiteit van Amsterdam, Amsterdam, The Netherlands; 2 Max Planck Institute for Human Cognitive and Brain Sciences, Department of Cognitive Neurology, Leipzig, Germany; 3 Research Center Juelich, Institute of Medicine, Juelich, Germany; University of California San Diego, United States of America

## Abstract

The option to choose between several courses of action is often associated with the feeling of being in control. Yet, in certain situations, one may prefer to decline such agency and instead leave the choice to others. In the present functional magnetic resonance imaging (fMRI) study, we provide evidence that the neural processes involved in decision-making are modulated not only by who controls our choice options (agency), but also by whether we have a say in who is in control (context). The fMRI results are noteworthy in that they reveal specific contributions of the anterior frontomedian cortex (viz. BA 10) and the rostral cingulate zone (RCZ) in decision-making processes. The RCZ is engaged when conditions clearly present us with the most choice options. BA 10 is engaged in particular when the choice is completely ours, as well as when it is completely up to others to choose for us which in turn gives rise to an attribution of control to oneself or someone else, respectively. After all, it does not only matter whether we have any options to choose from, but also who decides on that.

## Introduction

We employ the term *agency* to refer to the capacity of human beings to make choices and to enact those choices in the world. By the term *context*, in contrast, we refer to the circumstances under which agency is assigned in a decision process. That is, in some situations we might be inclined to give up agency and leave the choice to others (e.g., out of politeness, or when we are too tired to choose, or when the consequences of the choice options are complex or unknown). In other situations, we might be told by someone else whether or not to take up agency in a decision process (e.g., when our partner asks us to choose a pattern for the new bathroom tiles, or when the manager tells us that we will not have a say in the decision about the new office location). Hence, the context under which decisions are made can be differentiated into a *free context*, where we can choose to take up or decline agency in the decision process, and a *determined context*, where it is up to others to assign agency. Agency in a decision process can in turn be differentiated into *self-agency*, where we choose from different alternatives ourselves, and *external agency*, where someone else chooses for us.

The literature on the neural bases of decision-making has focused largely on the process of choosing per se (i.e., selecting an appropriate stimulus or action from several alternatives). This literature documents growing evidence for the crucial role of the medial frontal cortex (MFC) in choice processes within a determined context, i.e., when subjects are explicitly told by the experimenter whether they have a choice or not [1], [2]. The neural bases of the modulatory influences of agency and (especially) context, which are of central importance to human decision-making in social situations, remain largely unexplored thus far.

In recent reviews and meta-analyses, it has been proposed that the MFC can be parcelled into functional divisions associated with different cognitive processes [3]–[5]. Specifically, the anterior MFC (aMFC) has been linked to processes associated with mentalizing, that is, the ability to understand intentions and goals as entertained by others [4], [6]. This ability is especially important in situations where another person's decision influences our own courses of action and leaves us being under external control. A more posterior region of the MFC (pMFC) is activated in action selection and action monitoring [2], [3]. Moreover, a portion of the pMFC, the so-called rostral cingulate zone [RCZ; 7], [8], was identified to be crucially involved in selection processes, that is, in the selection of response sets [9], and task sets [10]. However, in these studies, selection processes were investigated in a context in which the choice options were designated by instructions. The present fMRI study was designed to provide a novel avenue for investigating the neural bases of more complex decision-making situations where the selection processes are modulated by both agency and context.

**Figure 1:**
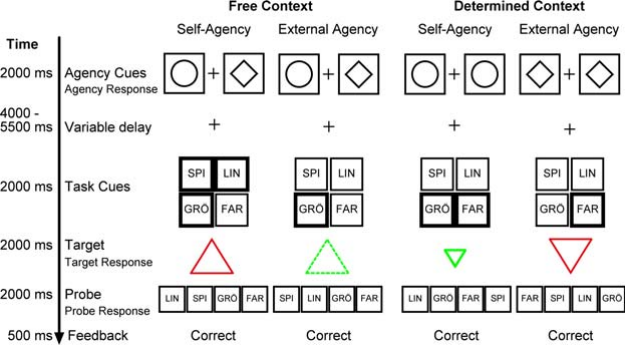
Paradigm. Schematic drawing of the trial sequence for free-context and determined-context trials. ‘Agency cues’ could either be circles (self-agency: participants choose the task themselves) or diamonds (external-agency: the task is chosen by the experimenter). In free-context trials, participants could choose to accept agency (by pressing the response button that was aligned to the circle cue in a spatially compatible mapping) or to decline agency (by pressing the response button aligned with the diamond cue). In determined-context trials, participants were either informed that agency was theirs (both agency cues were circles) or that agency was not theirs (both cues were diamonds). Agency cues were presented for 2000 ms and were followed by a variable interval of 4000–5500 ms. Subsequently, ‘tasks cues’ were presented in the 2×2 grid with German abbreviations ‘FAR’ (color), ‘SPI’ (orientation), ‘GRÖ’ (size), and ‘LIN’ (line). Quadrants with bold lines indicated that a task was available for choice (varied between 1–3 degrees of freedom). Target and probe stimulus were presented until a response was given or an interval of 2000 ms was exceeded. Finally, feedback was presented for 500 ms.

Trials in the fMRI experiment consisted of two parts, a first part where agency for the upcoming decision process was assigned, and a second part where a task was chosen and executed. In *free-context trials*, participants could decide whether they wanted to assume or decline agency in the subsequent decision process. More specifically, participants viewed two cue symbols on the screen, associated with self-agency and external-agency, respectively (Fig. 1). They could choose between accepting agency (i.e. choosing themselves which task to perform on the upcoming stimulus), or declining agency (i.e. leaving the to-be-performed task to be designated by instructions). In contrast, in *determined-context* trials, participants were told whether they had to perform as agents or not. This design allows us to address two important questions: (i) is the RCZ differentially engaged in self-agency decision-making, where the participants can choose a task themselves, compared to external-agency decision-making, where the experimenter determines the task; and (ii) is the aMFC differentially engaged in decision-making processes in free-context trials, where the participants can choose between self-agency and external-agency, compared to determined-context trials, where the experimenter decides about agency?

## Results and Discussion

To answer these questions, we computed a random-effects analysis of the fMRI data to test for the main effects of agency (self-agency vs. external-agency), context (free vs. determined), and their interaction. These analyses were computed with respect to two different onsets within the same trial, that were separated by a long and variable interval (mean duration = 4750 ms). The first onset was set to the presentation of the ‘agency cues’ (Fig. 1) at the beginning of each trial. The second onset was set to the ‘task cues’ where participants could choose between two or three tasks or had no choice (see Fig. 1). Thus, if participants decided themselves (self-agency on free-context trials) or were asked by the experimenter (self-agency on determined-context trials) *to choose*, then they had two or three degrees of freedom in task choice. When they decided or were told to leave the choice to the experimenter (external-agency on free-context trials, and determined-context trials, respectively), they had no freedom in task choice.

**Figure 2:**
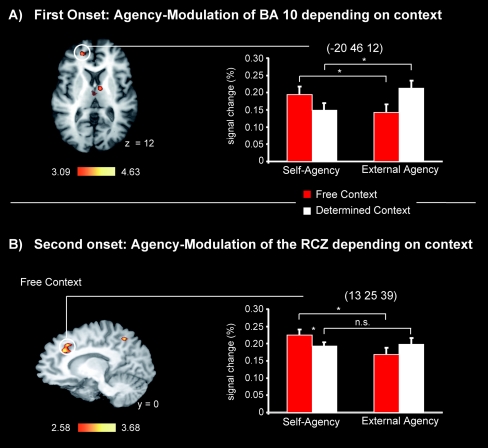
Activation maps averaged over 21 subjects mapped onto an individual brain. Red labels indicate positive z values. Coordinates are given in Talairach space. Error bars reflect standard errors. A) Activation elicited by the agency cues: Cross-over interaction between agency and context. Note that we also obtained a significant cross-over interaction in the mediodorsal nucleus of the thalamus (7, −5, 12). The activation in BA 10 comprised 107 contiguous voxels; the activation in the Thalamus comprised 103 contiguous voxels. B) Activation elicited by the task cues: Region of interest analysis in the rostral cingulate zone (RCZ) showing the main effect of agency (self-agency>external agency) only in free-context trials. The activation in the RCZ comprised 91 contiguous voxel.

The whole brain analysis of the fMRI data revealed that the agency cue and the task cue elicited distinct patterns of activation. First, for the agency cue onset, significant activations were obtained for the interaction between context and agency (see Fig. 2A) in the aMFC (identified as Brodmann Area (BA) 10 extending into BA 9). The main contrasts for agency (self-agency vs. external-agency) and context (free context vs. determined context) did reveal no significantly stronger activations for self-agency and free context.

Interestingly, statistics on the percent signal changes revealed a cross-over interaction (F(2,19) = 20.54, p<.001), with free-context/self-agency and determined-context/external-agency eliciting greater BA 10 activation than free-context/external-agency and determined-context/self-agency, respectively (t(20)>2.46, p<.05). This pattern replicates and extends the recent finding that this aMFC region is active in a determined context during externally guided (determined-context/external-agency) but not internally guided (determined-context/self-agency) action selection [9]. BA 10 has been implicated both in processing information about the self and in understanding the minds of others. More specifically, it is engaged consistently during a variety of self-referential tasks that require reporting one's own internal states, including adopting a first-person perspective [for a review see 11] as well as in processes related to perspective-taking and mentalizing [for a review see, e.g. 4]. More specifically, it has been proposed that this cortical region is involved in attending to onés own decision processes as well as in mentalizing about the decisions of similar others [11], [12]. The free-context/self-agency condition refers to choices made by oneself, whereas the determined-context/external-agency condition refers to choices determined by someone else. The crucial feature of these conditions is that there is a fit between the degrees of freedom in context and agency: Voluntarily deciding about agency might thereby give rise to a coherent attribution of control to oneself whereas having no say about agency is in line with a coherent attribution of control to someone else. The free-context/external-agency and determined-context/self-agency conditions are more ambivalent because either one decides to leave agency to others, or self-agency is imposed by others. Hence, these two conditions do not refer to a clear self/other perspective.

Second, for the task cue onset, the whole brain analysis of agency (self-agency vs. external-agency) revealed activation in the RCZ (see Fig. 2B). This activation site closely resembles the area active when participants voluntarily select between different task or response sets [9], [10]. Moreover, a subsequently performed region of interest (ROI) analysis confirms our prediction that context modulates the more basic decision-making processes: If participants decided to choose between different task sets (free context), then the RCZ was activated differentially for self-agency as compared to external-agency conditions, t(20) = 2.97, p = .007 (see Fig. 2B). Significantly higher percent signal changes were also obtained for the self-agency condition for the free compared to the determined context t(20) = 2.8, p = .01. In contrast, RCZ activation did not differ between self- and external-agency conditions in the determined context, t(20) = 0.24, p = .81. The interaction between agency and context nearly reached significance (F(2,19) = 3.8, p<.06). Finally, the activation did not differ between the DF3 and DF2 condition.

In a previous study [10], RCZ activation was more pronounced in self-compared to external-agency conditions. In that study, the self-agency condition was the condition that provided maximum freedom of choice. In the present study, maximum freedom of choice was obtained in self-agency conditions in free-context trials. Thus, while the RCZ was found activated in a determined context also (see Fig. 2B), the self-agency condition in free context trials offered the most degrees of freedom in choice and hence revealed the highest RCZ activation. We therefore argue that activation in the RCZ is modulated by both, context and agency.

In a final analysis, we aimed to show that the aMFC and the RCZ reveal distinct roles in decision-making. Hence, we computed two three-way interactions separately for both onsets for agency, context, and ROI. Please note that the percent signal changes from the aMFC and RCZ were derived from neutral contrasts (see Supplementary Materials S1). The analysis revealed a significant agency×context×ROI interaction for the first onset, (F(2,19) = 5.14, p<.05), and a marginally significant agency×context×ROI interaction for the second onset (F(2,19) = 3.85, p = .06). For the first onset, the aMFC revealed again a significant crossing-over interaction for agency and context (F(2,19) = 14.32, p = .001), which was not apparent in the RCZ. Moreover, for the second onset, the RCZ was relatively stronger activated for the self-agency compared to the external-agency conditions in the free context (t(20) = 1.86, p = .07), but not in the determined context (t(20) = 1.05, p = .36). An opposite pattern was found for the aMFC, where relatively stronger activation was revealed for the external-agency conditions compared to the self-agency condition in the free context (t(20) = 2.62, p = .01), but not in the determined context (t(20) = .16, p = .87). However, computing two-way interactions for the RCZ and aMFC for the second onset did not yield significant results (F<1).

In conclusion, the present results provide evidence for the vital roles of context and agency in modulating decision-making processes. The findings are noteworthy in that they reveal specific contributions of the aMFC (*viz*. BA 10) and the RCZ in decision-making processes. The RCZ is engaged when conditions clearly present us with the most choice options. The BA 10 is engaged in particular when the choice is completely ours, as well as when it is completely up to others to choose for us. After all, it does not only matter whether we have any options to choose from, but also who decides on that.

## Materials and Methods

### Participants

21 healthy volunteers were recruited. We obtained written consent from all 21 participants, 12 females and 9 males (age: mean = 26.05, SD = 2.35), prior to the scanning session. All subjects had normal or corrected-to-normal vision. No subject had a history of neurological, major medical, or psychiatric disorder. All participants were right handed as assessed by the Edinburgh Inventory [13].

### Behavioral Task

The design consisted of two independent variables, ‘context’ (free-context trials vs. determined-context trials) and ‘agency’ (self-agency vs. external-agency). Moreover, the degrees of freedom in choice in the self-agency condition varied between 2 and 3 (DF2 and DF3). The ‘agency cues’ could either be circles or diamonds, associated with either ‘self-agency’ or ‘external-agency’ respectively (see Fig. 1). At the beginning of each trial, two circles or two diamonds (both conditions referring to a determined-context trial) or the circle together with the diamond (referring to a free-context trial) were presented for 2000 ms. In the condition with two different cues, the presentation side of the cues was counterbalanced within subjects. In both free-context and determined-context trials, participants were required to give a response with the index or middle finger of the right hand. For the determined-context trials, the response button press had no consequence compared to the free-context trials where participants indicated with a spatially compatible mapping whether they accepted or declined agency in the secondary task. Note that participants had to select a specific response button in all conditions so that mere response selection processes could not account for the differential activation between free-context and determined-context trials. After a variable delay of a mean duration of 4750 ms, four cues each associated with one of four simple discrimination tasks, i.e. color, orientation, size, and line task, were presented [10]. The cues were foveally presented in a 2×2 grid with a visual angle of 2.8° (Fig. 1). Each quadrant of the grid contained a semantic abbreviation for one of the four tasks, i.e. ‘FAR’ for ‘Farbe’ which corresponds to color, ‘SPI’ for ‘Spitze’ which corresponds to orientation, ‘GRÖ’ for ‘Groesse’ which corresponds to size, and ‘LIN’ for ‘Linie’ which corresponds to line. The locations of the cues were balanced across participants. There were 16 possible ‘cue-location mappings’ which were randomly assigned to the participants. In order to instruct the participants which task could be chosen, the quadrants where either bold, indicating that this task could be chosen, or not, indicating that this task was not available for choice. After a constant interval the target (a triangle) was presented. This target was multivalent, i.e. the target contained one value of each discrimination task (i.e. one out of two colors, one out of two orientations, one out of two sizes and one out of two line types) resulting in 16 possible values of a target. Corresponding to their choice of task, participants had to respond to the target with either the index or middle finger of the right hand. In order to check for accuracy and to give valid feedback, the four semantic abbreviations of the four tasks were presented again. This time, they were horizontally aligned while the locations of the semantic cues were pseudo-randomized, assuring that participants could not prepare for this response during the second decision time. At this point, participants were required to indicate which task they had actually chosen and responded to with their first response by pressing the spatially compatible key with the fingers of their left hand. Finally, a feedback for wrong, missed, or correct responses was presented.

We also included 24 catch trials to make sure that participants engaged in the selection of agency at the presentation of the agency cues. These trials were highly comparable to the experimental trials. The only difference pertained to the presentation of match cues instead of the target and probe stimuli. The match cues consisted of 2 words, ‘stimmt’ (i.e. match) or ‘stimmt nicht’ (i.e. no match) which were presented next to each other in the middle of the screen. The presentation side of the match cues was pseudo-randomized. Participants had to indicate with their right index or middle finger with a spatially compatible mapping whether the agency option (i.e., self-agency vs. external-agency) presented at the beginning of a trial matched the degrees of freedom of tasks presented in the 2×2 grid. There were 6 different conditions for the factors context, agency and match which were all counterbalanced within subjects. Finally, also 10 null events were included, which were pseudo-randomly interspersed. The null events were introduced to compensate for the overlap of the blood-oxygenation level dependent (BOLD) response between adjacent trials.

### Trial timing

The timing of the sequence of trials was triggered from the magnetic resonance imaging (MRI) control every 18 seconds. The trials started with a variable oversampling interval of 0, 500, 1000 or 1500 ms to obtain an interpolated temporal resolution of 500 ms. Then, a fixation cross was presented for 500 ms followed by the agency cues which were presented for 2000 ms. After the presentation of the agency cues, a variable delay of 4000–5500 ms with a variable oversampling interval of 0, 500, 1000 or 1500 ms was presented. Subsequently, the 2×2 grid appeared for 2000 ms. Then the target was presented until a response was made with the right hand or until the response interval exceeded 2000 ms. After the first response was given, the probe stimulus was presented. It remained on the screen until the second response was made with the left hand or until the response interval exceeded 2000 ms. Finally, valid feedback was presented for 500 ms.

The experiment lasted for about 60 min and consisted of one block starting out with two dummy trials which were excluded from further analysis. In total, 100 experimental trials (without catch trials and null-events) were presented, resulting in 25 trials for each the determined self-agency and determined external-agency condition and 25.75 trials for the free self-agency and 23.66 trials for the free external-agency condition. Even though it was not possible to control for the frequencies of self-agency or external-agency in the free-context trials, we pre-selected participants who chose both options approximately equally frequent (see also section ‘pre-selection of participants’). A comparable constraint applied to the presentation of the degrees of freedom (DF2 and DF3) in the self-agency condition. There were 14 different combinations of DF and the four discrimination tasks (DF1 = 4 combinations; DF2 = 6 combinations; DF3 = 4 combinations). Again, these could not be introduced in a pre-randomized task sequence due to the manipulation of self-agency. Therefore, subsets of each combination of DF and tasks were defined. Trials were pseudo-randomly chosen from each subset, thereby assuring that each combination was presented with approximately equal frequency. Furthermore, it is important to note that the trial following the DF3 condition was not constrained to the one task which was not valid for choice before.

### Pre-selection of participants

Before participants entered the scanner they underwent a training session of 30 min in which they were familiarized with the task. On average this training session took place 2 days before the scanning session. In the training session and also before the scanning procedure the participants were instructed to engage in every trial and consciously think about their choice on each trial. Please note that participants were not instructed to counterbalance between conditions or were otherwise biased to perform a specific selection strategy. Therefore, the training session was included to select only those participants who assumed and declined agency approximately equally often in the free-context trials and also showed no bias in selecting a specific response button towards the agency cues (see Supplementary Materials S1). Finally, 21 out of 28 participants taking part in the training session were included in the fMRI study.

### Magnetic resonance imaging scanning procedure

The experiment was carried out on a 3T scanner (Medspec 30/100, Bruker, Ettlingen, Germany). 20 axial slices were acquired (19.2 cm field of view, 64×64 matrix, 4 mm thickness, 1 mm spacing) parallel to the AC-PC plane and covering the whole brain. Slice gaps were interpolated to generate output data with a spatial resolution of 3×3×3 mm. We used a single shot, gradient recalled echo planar imaging (EPI) sequence (repetition time 2000 ms, echo time 30 ms, 90° flip-angle). Prior to the functional runs, correspondingly 20 anatomical MDEFT slices and 20 EPI-T1 slices were acquired. Stimuli were displayed using a head-mounted mirror-system.

### Functional magnetic resonance imaging analysis

Analysis of functional magnetic resonance imaging (fMRI) data was performed using the inhouse LIPSIA software [14]. First, functional data were corrected for movement artifacts. The temporal offset between the slices acquired in one scan was then corrected using a sinc interpolation algorithm. Data were filtered using a spatial Gaussian filter with sigma = 0.8 (this refers to a FWHM = 5.65 mm). A temporal high-pass filter with a cutoff frequency of 1/160 Hz was used for baseline correction of the signal. All functional data sets were individually registered into three-dimensional (3D) space using the participants̀ individual high-resolution anatomical images. This 3D reference data set was acquired for each participant during a previous scanning session. The two-dimensional anatomical MDEFT slices, geometrically aligned with the functional slices, were used to compute a transformation matrix containing rotational and translational parameters that register the anatomical slices with the 3D reference T1 data set. These transformation matrices were normalised to the standard Talairach brain size [15] by linear scaling and finally applied to the functional data. The statistical evaluation was performed using the general linear model for serially autocorrelated observations [16]. The design matrix was generated with a synthetic hemodynamic response function [17] and its first derivative. The onsets for the event-related analysis were set to the presentation of the agency cues at the beginning of each trial and to the 2×2 grid. The model equation was convolved with a Gaussian kernel with a dispersion of 4 sec full width at half maximum. Contrast maps were generated for each participant. After the individual functional datasets were all aligned to the same stereotactic reference space, a group analysis was performed. A one-sample *t* test of contrast maps across participants (random-effects model) was computed to ascertain whether observed differences between conditions were significantly different from zero. Subsequently, *t* values were transformed into z-scores. To correct for false-positive results, in a first step, an initial voxelwise z-threshold was set to Z = 2.58 (p = 0.005, uncorrected) for the agency×context interaction contrast (100 trials) and to Z = 2.33 (p = 0.01, uncorrected) for the conditional main effect of agency in the free context (50 trials). In a second step, the results were corrected for multiple comparisons using cluster-size and cluster-value thresholds obtained by Monte-Carlo simulations using a significance level of p = 0.05. To ensure that the reported activations, namely the MFC and RCZ, are significantly activated at p<.05 (corrected at the cluster-level), a cluster-size of 1620 mm^3^ (60 contiguous 3×3×3 mm voxels) for the interaction between agency×context and a cluster-size of 2439 mm^3^ (90 contiguous voxels) for the conditional main effect of agency in the free context was required. In total we could ensure, that the reported activations we focus our discussion on, namely the aMFC and the RCZ, are significantly activated at p<.05, corrected for multiple comparisons at the cluster-level.

To compute the percent signal change of the hemodynamic response in the aMFC, all significantly activated voxels exceeding the critical threshold in the group-averaged whole-brain interaction between context×agency and belonging to a contiguous cluster in the aMFC were included. Analogously, we also computed the percent signal change of the hemodynamic response in the RCZ including all significantly activated voxels that exceeded the critical threshold in the group-averaged main effect of agency and belonged to a contiguous cluster in the RCZ. We then extracted the time course of the signal underlying these activated voxels for each participant from the preprocessed data. The percent signal change was calculated in relation to the mean signal intensity across all timesteps for these voxels. The signal change was averaged for each condition for 16 seconds beginning with the presentation of the agency cues and the presentation of the 2×2 grid, respectively. We then subtracted the time course of the null event from the time course of the relevant conditions to compensate for the overlap of the BOLD response [18]. The logic is that null events are (at least on average) embedded within the same past and future trial conditions as a regular event, and thus have the same preceding and succeeding average BOLD signal. By subtracting the null event from the relevant condition, one assumes that the brain area, i.e. activated voxels, exhibit no activation for the null event so that the remaining BOLD signal is solely due to the experimental manipulation. We searched for the largest value of the signal in the time window between 4 to 8 seconds after the presentation of the agency cues and the 2×2 grid, respectively. Finally, these values were averaged across participants.

To test whether the aMFC and the RCZ reveal distinct functional roles in decision-making processes, additional region of interest (ROI) analyses with unbiased ROIs were conducted. In a first step, neutral contrasts, i.e. all conditions vs. resting baseline, were computed separately for both onsets. Based on these contrasts, in a second step, ROIs were generated for the aMFC [3] and the RCZ [2], [7]. The ROI for the aMFC was generated from the neutral contrast, i.e. all conditions vs. resting baseline, for the first onset, and defined as a sphere with 3 mm radius centered on the peak voxel of the activation (see Supplementary Materials S1). The same procedure was applied to generate the ROI in the RCZ. Here the neutral contrast, i.e. all conditions vs. resting baseline, for the second onset was taken, and the ROI was centered at the peak voxel of the activation, with a sphere radius of 3 mm (see Supplementary Materials S1). In a third step, the percent signal change from each ROI, i.e. aMFC and RCZ, was extracted separately for each subject, for each condition, and for each onset. Finally, these values were subjected to repeated measures ANOVAs to statistically test for three-way interactions between agency, context, and ROI, separately for the first and second onset.

## Supporting Information

Supplementary Materials S1 (0.04 MB DOC)

Figure S1 (10.37 MB PNG)
