# Supplementary material for: When the Choice Is Ours: Context and Agency Modulate the Neural Bases of Decision-Making
Source: PLoS One. 2008 Apr 2;3(4):e1899. doi: 10.1371/journal.pone.0001899 (PMC2290971; doi:10.1371/journal.pone.0001899)
Supplement: Supplementary Materials S1 — (0.04 MB DOC) [file pone.0001899.s001.doc]

**Supplementary Material Text S1**

###### Results

Behavioral performance

For the analyses of error rates and reaction times (RTs) to the target, data were submitted to a repeated measures analysis of variance (ANOVA) with the independent variables context (free-context trials vs. determined-context trials) and agency (self-agency (DF2 and DF3) vs. external-agency (DF1)). The overall accuracy was 96.4%. The error rates revealed no significant effects (*t*s < 1). The overall accuracy was also computed for the catch trials revealing 89.3% of correct trials. Again, the error rates revealed no significant effects (*ts* < 1).

The RTs for the target response only revealed a significant main effect for agency, F(2,19) = 3.58; p < .05, indicating prolonged RTs for the self-agency conditions compared to the external-agency condition. Subsequent paired t-tests revealed significant differences for the DF2 condition vs. the DF1 condition (860 ms vs. 804 ms), *t*(20) = 3.01; p < .01, and the DF3 condition vs. the DF1 condition (853 ms vs. 804 ms), *t*(20) = 2.39; p < .05, indicating higher RTs for the self-agency conditions compared to the external-agency conditions (DF2 and DF3 = 856 ms vs. DF1 = 804 ms). There was no significant influence of the degrees of freedom within the self-agency conditions (DF3 vs. DF2), *t*(20) = .52; p > .60. Since participants were instructed that the response to the agency cues and the probe stimuli were unspeeded and the response to the latter one only relevant for giving valid feedback, the RTs were not analyzed.

Selection strategies

Since we tried to equalize the frequency of task types in combination with the degrees of freedom, it is important to note that the interpretation of specific selection strategies is highly constrained. However, to test for the potential occurrence of specific selection strategies, several post-hoc analyses were performed with respect to the first onset, i.e. agency cues, and the second onset, i.e. task cues. For the first onset, the dependent measure was the number of response buttons that were chosen both in the free and determined task context and the number of agency types that were chosen in the free task context to account for preferences in selection strategies.

One might argue that participants preferred a specific response button instead of actively engaging in the task on every trial. Hence, we analyzed the frequencies of chosen response buttons to the agency cues independent of the free or determined context. The results revealed no significant differences between the number of right or left response button presses towards the agency cues (*t*s < 1) indicating that there was no general bias in selecting a specific response button. The same analysis was also conducted separately for the free and determined context condition showing that there was no bias in selecting one over another response button. Moreover, we were interested in specific selection strategies in the free context condition. Hence, we tested whether there was a bias in selecting the self- or external-agency condition, respectively. Again, no significant difference was found between the frequencies of self- and external-agency condition in the free context (*t*s < 1).

For the second onset the dependent measure was the number of trials that were chosen for each particular task. The independent variables were task type, to account for a preference in ‘task selection’, and cue location to account for a preference in ‘location selection’. Both analyses revealed no significant effect (*t*s < 1). Moreover, to account for task selection strategies, an additional ANOVA with the dependent measure RTs to the target for the three-level factor DF and four-level factor discrimination tasks were computed. The results revealed a significant main effect for the degrees of freedom F(2,19) = 4.27; p < .05, which is in line with the finding that the self-agency conditions revealed prolonged RTs compared to the external-agency condition (see previous section). However, there was neither a significant main effect for the discrimination tasks nor a significant interaction (*t*s < 1).

Finally, it is important to note that participants always had to switch between tasks so that the task that was chosen on the present trial was never valid for choice on the next trial. Importantly, the trial following the DF3 condition was not constrained to the one task which had not been valid for choice before.

Figure Legends

Fig. S 1.

Additional analyses with unbiased ROIs derived from neutral contrasts (all conditions vs. resting baseline trials separately for each onset). Shown are spheres of 3 mm centered on the peak cluster-level corrected voxel in the posterior part of the aMFC (-14,36,3) and RCZ (7,15,45). Bar graphs depict the difference in percent signal change for self-agency vs. external-agency for the free and determined context separated for the first and second onset. Error bars indicate the standard errors.
