# Supplementary figures and images for: When the Choice Is Ours: Context and Agency Modulate the Neural Bases of Decision-Making
Source: PLoS One. 2008 Apr 2;3(4):e1899. doi: 10.1371/journal.pone.0001899 (PMC2290971; doi:10.1371/journal.pone.0001899)

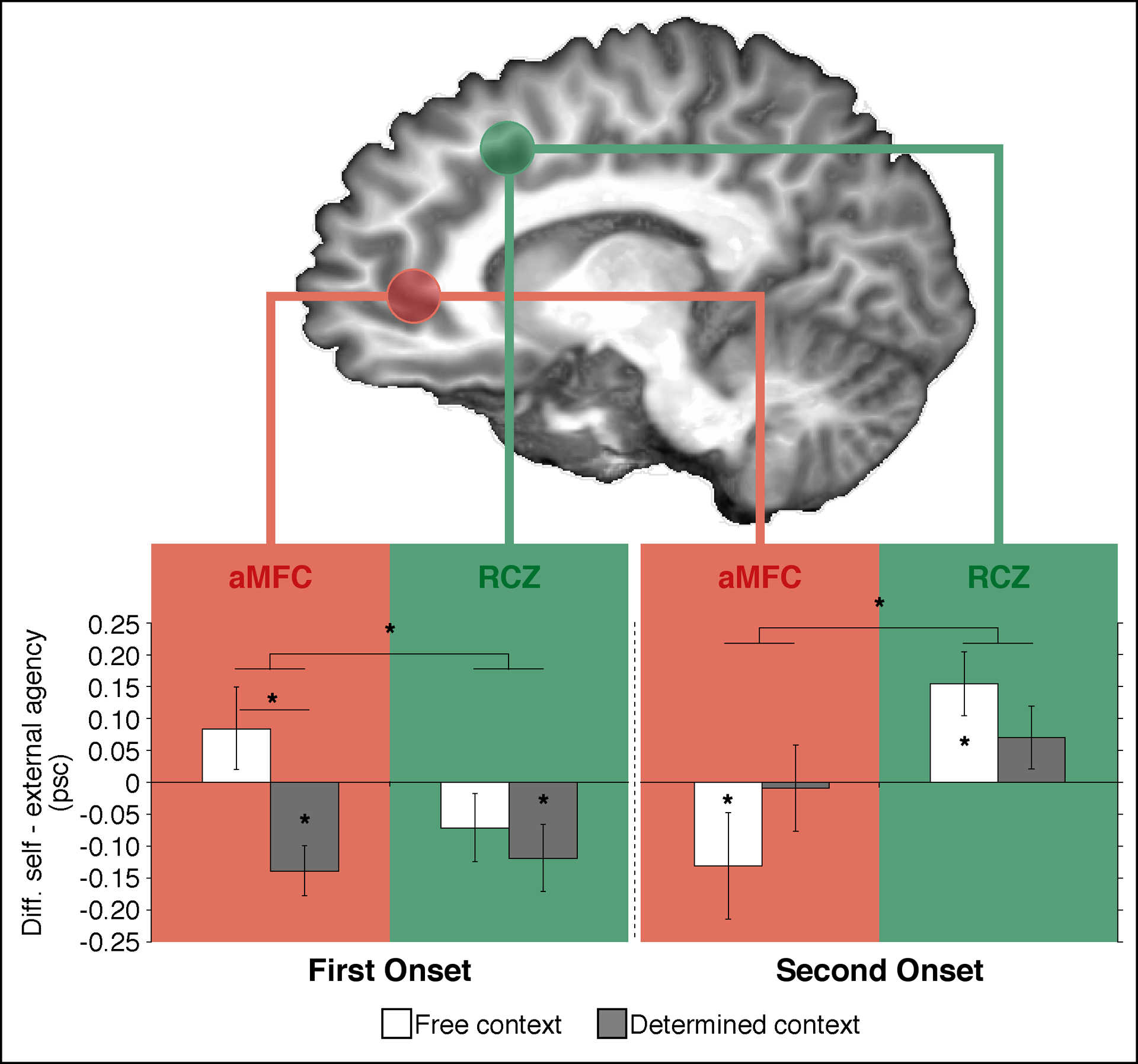

Supplement: Figure S1 — (10.37 MB PNG) [file pone.0001899.s002.png]
